# Allele frequency net 2015 update: new features for HLA epitopes, KIR and disease and HLA adverse drug reaction associations

**DOI:** 10.1093/nar/gku1166

**Published:** 2014-11-20

**Authors:** Faviel F. González-Galarza, Louise Y.C. Takeshita, Eduardo J.M. Santos, Felicity Kempson, Maria Helena Thomaz Maia, Andrea Luciana Soares da Silva, André Luiz Teles e Silva, Gurpreet S. Ghattaoraya, Ana Alfirevic, Andrew R. Jones, Derek Middleton

**Affiliations:** 1 Institute of Integrative Biology, University of Liverpool, Liverpool, UK; 2 Center for Biomedical Research, Faculty of Medicine, Autonomous University of Coahuila, Torreon, Mexico; 3 Human and Medical Genetics, Institute of Biological Sciences, Federal University of Pará, Brazil; 4 Department of Molecular and Clinical Pharmacology, Institute of Translational Medicine, University of Liverpool, Liverpool, UK; 5 Transplant Immunology Laboratory, Royal Liverpool and Broadgreen University Hospital, University of Liverpool, UK; 6 Institute of Infection and Global Health, University of Liverpool, UK

## Abstract

It has been 12 years since the Allele Frequency Net Database (AFND; http://www.allelefrequencies.net) was first launched, providing the scientific community with an online repository for the storage of immune gene frequencies in different populations across the world. There have been a significant number of improvements from the first version, making AFND a primary resource for many clinical and scientific areas including histocompatibility, immunogenetics, pharmacogenetics and anthropology studies, among many others. The most widely used part of AFND stores population frequency data (alleles, genes or haplotypes) related to human leukocyte antigens (HLA), killer-cell immunoglobulin-like receptors (KIR), major histocompatibility complex class I chain-related genes (MIC) and a number of cytokine gene polymorphisms. AFND now contains >1400 populations from more than 10 million healthy individuals. Here, we report how the main features of AFND have been updated to include a new section on ‘HLA epitope’ frequencies in populations, a new section capturing the results of studies identifying HLA associations with adverse drug reactions (ADRs) and one for the examination of infectious and autoimmune diseases associated with KIR polymorphisms—thus extending AFND to serve a new user base in these growing areas of research. New criteria on data quality have also been included.

## INTRODUCTION

The Allele Frequency Net Database (AFND) was designed to provide a free centralized resource for the storage of frequencies on the polymorphisms of several immune-related genes (1). The website contains information primarily on the frequencies of several genes from the human leukocyte antigens (HLA) system, killer-cell immunoglobulin-like receptors (KIR), major histocompatibility complex class I chain-related genes (MIC) and a number of cytokine gene polymorphisms. These loci are among the most polymorphic in humans and play key roles in the immune system response, as well as being important for donor-recipient matching in organ and stem cell transplantation success (2,3). These loci have also been studied extensively due to associations between polymorphisms and response to infectious diseases (4) or susceptibility to autoimmune diseases (5–7). Recently, there is also a growing field of study identifying associations between particular HLA polymorphisms and increased risk for adverse drug reactions (ADRs) (8,9). The HLA region is also commonly analyzed in anthropology studies (10). The HLA system comprises more than 20 genes, however, only six loci are routinely typed by laboratories, i.e. HLA-A, -B, -C for Class I and HLA-DRB1, -DQB1 and -DPB1 for Class II. Hence, most of the data sets in AFND cover principally these genes, also known as classical HLA loci. At present, more than 11 000 HLA class I or II alleles have been reported at the IMGT/HLA database (Release 3.17.0.1, August 2014) (11).

The first release of AFND in 2003 consisted of only a few sections, and frequencies of HLA alleles/allelic lineages were shown in static web pages. In 2008, the database was substantially re-developed, producing the system described in a previous publication in the 2011 database issue of *Nucleic Acid Research* (1), which readers should consult for a detailed description of the purpose and background to the system. Since then, the database has grown substantially in terms of the number of populations covered and the number of users/citations. In the past 3 years, more than 75 000 different users from 172 countries accessed the database. In this article, we describe new population data added, new developments in validating the quality of data sets in AFND, as well as new sections for capturing frequency data on ‘HLA epitopes’ (structure-level polymorphisms recognized by antibodies), associations between KIR polymorphisms and disease and associations between HLA alleles and ADRs that have been identified from the literature.

## DESCRIPTION OF AFND AND SOURCES OF DATA

The database of AFND is currently implemented in MS SQL Server in the latest release (previously maintained in MySQL). Web pages are constructed using active server pages, Javascript and AJAX technology to improve user interaction and data visualization.

### Normal population data

AFND receives data from three main sources: (i) data from peer-reviewed publications, (ii) from populations that are analyzed at International HLA and Immunogenetics Workshops (IHWS) and (iii) submissions from individual laboratories across the world. However, by far the most data (80%) come from data extraction and curation by the AFND team from peer-reviewed publications. As such, a vast amount of data may be missing, which, although of good quality, is not published and we encourage labs with such data to contact us. The literature review comprises not only histocompatibility- and immunogenetics-related journals, but also, we have established semi-automated methods using regular structured queries of literature databases to verify other journals that may contain suitable data for inclusion.

**Figure 1.**
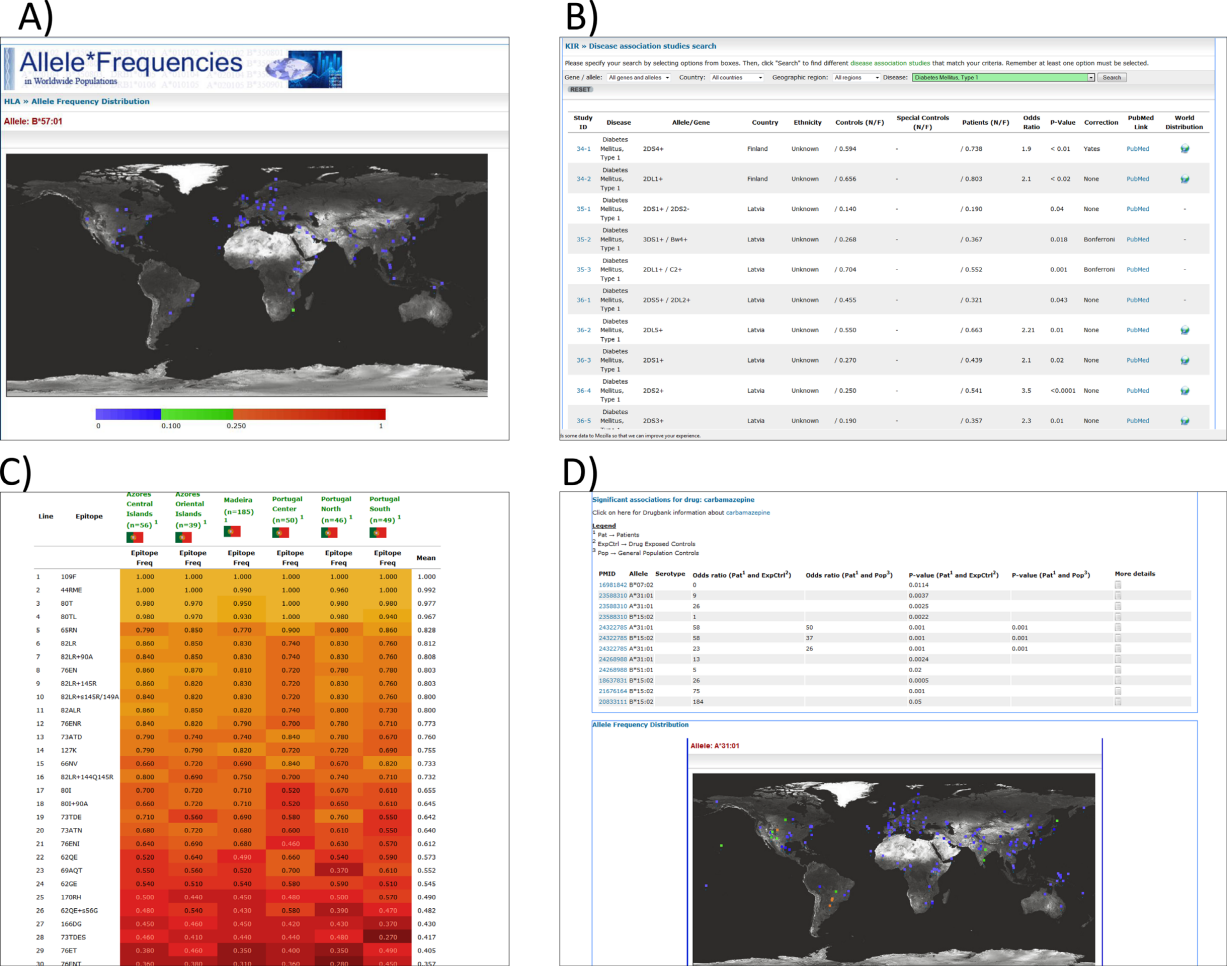
(**A**) A world map in AFND showing the global allele frequencies of HLA B*57:01, which, for example, has been associated with adverse reactions to abacavir; (**B**) a query of KDDB for populations/studies in which associations between KIR genotypes have been made with Type 1 diabetes; (**C**) a heat map view of several populations in the HLA epitope database (filtered by epitopes from ‘locus A+B’); (**D**) a drug report in AFND showing all association data for carbamazepine.

As of September 2014, we have collected information on >1400 healthy populations from more than 10 million people. The HLA section contains the majority of the submissions with 1022 populations, followed by populations analyzed for polymorphisms in KIR (229), cytokine genes (114) and MIC (60) (Table 1, figures correct in September 2014). Currently, data sets from 138 countries are included within AFND—with highest coverage (by population number) the United States (121 populations), followed by China (110 populations)—summarized under the ‘Populations-Pops By Region’ menu in the database. In terms of the number of individuals, United States, Brazil and Italy have the largest amount of data, due to the inclusion of large data sets from bone marrow donor registries in the database. As described previously (1), the most popular tools in AFND include queries for particular allele/haplotype frequencies (viewed as a table or world map—Figure 1A), or analysis of all allele/haplotype frequencies within a given population or geographic region of the world.

**Table 1.** Frequency data sets by polymorphic region at AFND, figures correct in September 2014

Polymorphic region	Population studies	Gene/allele data	Haplotype data	Genotype data
HLA	1022	1004	370	-
KIR	229	228	-	146
Cytokine	114	114	-	-
MIC	60	60	21	-
Totals	1425	1406	391	146

Although all submissions by contributors are considered for inclusion, AFND has introduced minimal criteria before the population becomes publicly accessible on the website. These minimum requirements include the homogenization on the naming of the populations, an appropriate assignment of the geographical region to which the population belongs, validation of frequency data such as ensuring allele names comply with the official nomenclature guidelines as described at http://allelefrequencies.net/quality.asp. These guidelines will continue to develop and be implemented across all data sets newly added to AFND.

### HLA epitope database

The presence of anti-HLA donor-specific antibodies in transplant patients is a crucial factor related to tissue and graft rejection. These antibodies target specific regions of HLA proteins that are different from the transplant patient's HLA proteins—termed ‘HLA epitopes’. Current efforts in matching for kidney transplantation minimizes the number of HLA antigen mismatches (very rarely a perfect match is achieved), yet this matching disregards structural differences (or similarities) between HLA proteins. As this concept is starting to get more recognition, and epitopes are being systematically defined (12), we developed a new section within AFND called Epitope Frequency Database (EpFreq-DB), for the storage of HLA epitope frequencies (the percentage of the population expressing a given epitope) across worldwide populations.

Two sources of data were used to generate HLA epitope frequencies: (i) HLA haplotype frequency data from AFND and (ii) HLA raw genotyping data, in both cases using data sets with at least 4-digit resolution (e.g. A*01:01). In this context ‘raw data’ is the genotype, comprising one or two alleles called at each HLA locus, of every individual in the population. Low-resolution data (2 digits) can encompass alleles with differences in the protein sequence, and thus epitopes cannot be unambiguously determined. Allele frequency data sets cannot be used for accurate inference of HLA epitope frequencies because the same epitope can be present in alleles at different HLA loci of the same individual, e.g. epitopes shared between some combination of HLA-A, B and C genes.

For calculating epitopes frequencies, two different methods were used according to the data type. From HLA raw genotyping data, they were calculated by counting individuals having at least one allele expressing a given epitope. The number of individuals expressing the epitope is then divided by the population sample size. From HLA haplotype frequency data, the Hardy–Weinberg equilibrium calculation (*p*^2^ + 2*pq* + *q*^2^) was applied to estimate epitope frequencies, treating a haplotype as expressing a given allele *p* or not *q*. The method has been extensively validated, and produces highly accurate estimates of epitope frequencies (e.g. *r*^2^ ∼ = 0.99 versus estimates from raw data). A full description of the methodology will follow in a subsequent manuscript.

To date, the HLA Epitope Frequencies Database (EpFreq-DB) comprises HLA class I epitope frequencies from 41 worldwide populations (17 raw/genotype data sets and 24 haplotype data sets) comprising more than 36 000 individuals. The definition/nomenclature of HLA epitopes and corresponding HLA alleles used in EpFreq-DB is based on the HLA Epitope Registry (http://www.epregistry.ufpi.br/) (12). EpFreq-DB has a query page for analyzing the epitope frequencies according to a number of different criteria or filters, displaying results as a tabular view, projected onto world maps for single epitopes, or as a comparison of epitope frequencies in different populations as a heatmap (Figure 1C).

### KIR and diseases

The section for KIR now includes a recently developed section (KIR and Disease Database—KDDB) containing associations that have been identified in the literature between KIR polymorphisms and disease—for detailed discussion and methods see (13). The development of KDDB was initiated, since there is a growing area of research demonstrating that the KIR genes carried by an individual can increase or decrease risk/severity of auto-immune and infectious diseases. However, many studies have relatively small sample sizes and different studies have conflicting findings. The development of KDDB enables researchers to examine all the published studies in one place, for example to foster meta-analyses and determine if findings in one study have been confirmed elsewhere.

Currently, KDDB has a total of 1179 KIR disease-association determinants captured from 204 articles, including those with single KIR genes, profiles of combined KIR genes and/or HLA class I ligands, and full KIR genotypes. According to the present database, KIR associations of 79 different disease terms have been included, of which 19 associations can be classified as infectious diseases, 32 as autoimmune or idiopathic diseases, three related to pregnancy, 16 to cancer, eight to chronic inflammatory diseases and one mental disorder. The web interface allows users to query KIR and disease associations applying several filters related to population demographics, disease studies and gene features. From the same location, the user can access links to submit new studies to the database, for example including those studies in which no association has been found—which are difficult to publish (and thus otherwise contribute to publication bias).

### ADR database

One of the biggest problems faced by clinicians and the pharmaceutical companies is the risk that patients might experience ADRs upon exposure to a drug treatment. Approximately 10% of all ADRs are immune mediated (14) and the most significant genetic associations have been related to HLA alleles (8). Given the huge inter-individual variability in HLA alleles only a small number of individuals are reported in each study leading to statistical analysis with low power. To assist the HLA and pharmacogenetic community, we have collated data sets from the literature, and they can be queried alongside the large data collection for normal populations within AFND at the allele and haplotype level. This provides a resource that not only facilitates meta-analyses but also enables users to examine the quality of published studies by comparing the frequencies of HLA alleles in ‘control’ cohorts with worldwide populations.

A similar curation protocol to KDDB was followed. Two inclusion criteria were used: first, the included studies utilized a case-control design, which provided statistical evidence for the association; second, high-resolution HLA typing was performed to generate data (low-resolution data are only present in the database for studies that performed both low- and high-resolution typing). Low-resolution data sets may be included in a later release of the database if we see demand from users for their inclusion. We included information on ethnicity, drug of interest and proportion of cases and controls that carry the HLA allele implicated in ADRs. Associations with >20 different drugs are captured in the current beta release of the database, with anti-epileptic drug carbamazepine having the most studies included. The aim of this new feature of AFND is under active development, and we aim to cover all published studies, and, as such, the amount of data included and the query tools provided will increase over the coming years. We have developed a feature—called ‘Drug reports’ highlighting all known associations for a given drug (Figure 1D). Users can see the worldwide distributions for the implicated alleles and haplotype data (from healthy data sets in AFND) and links out to IMGT/HLA for sequence alignments of the implicated alleles.

## FUTURE DEVELOPMENTS

Future challenges and plans for AFND include improving the direct data submission process for all sections within AFND, for example by a direct connection with the Human Immunology journal as part of the manuscript submission process. We will also continue to develop quality control tools, for example developing a ‘gold standard’ set of populations in AFND that meet high quality/validation criteria. We also plan to develop the ability to perform statistical analyses and visualization of population data on the AFND site to facilitate users and as an additional mechanism for stimulating direct data submissions. To do this we will be asking that submissions include raw data.

## CONCLUSION

AFND is the most comprehensive database for frequency data, relating to immune genes/alleles in worldwide populations. AFND receives ∼300 hits per day, and is widely cited in a variety of different clinical and research fields. The origins of the database were to provide support for the Histocompatibility and Immunogenetics community, in understanding worldwide distribution of HLA genotypes. The database has continued to support these fields, while developing new features (such as HLA epitope frequencies) that provide a new viewpoint on these highly complex data sets. AFND has recently expanded to support new research groups, particularly those working on auto-immune disorders and infectious diseases, for which associations with KIR polymorphism are increasingly being identified. AFND is also developing new features to support pharmacogenetic research, since there is a rapidly growing field emerging, as it becomes increasingly clear that HLA molecules play a role in many ADRs.

## AVAILABILITY

AFND Homepage: http://www.allelefrequencies.net

Contact: support@allelefrequencies.net
